# Complete Genome Sequence of the N_2_-Fixing Broad Host Range Endophyte *Klebsiella pneumoniae* 342 and Virulence Predictions Verified in Mice

**DOI:** 10.1371/journal.pgen.1000141

**Published:** 2008-07-25

**Authors:** Derrick E. Fouts, Heather L. Tyler, Robert T. DeBoy, Sean Daugherty, Qinghu Ren, Jonathan H. Badger, Anthony S. Durkin, Heather Huot, Susmita Shrivastava, Sagar Kothari, Robert J. Dodson, Yasmin Mohamoud, Hoda Khouri, Luiz F. W. Roesch, Karen A. Krogfelt, Carsten Struve, Eric W. Triplett, Barbara A. Methé

**Affiliations:** 1 J. Craig Venter Institute, Rockville, Maryland, United States of America; 2 Department of Microbiology and Cell Science, University of Florida, Gainesville, Florida, United States of America; 3 Department of Bacteriology, Mycology and Parasitology, Statens Serum Institut, Copenhagen, Denmark; University of Toronto, Canada

## Abstract

We report here the sequencing and analysis of the genome of the nitrogen-fixing endophyte, *Klebsiella pneumoniae* 342. Although *K. pneumoniae* 342 is a member of the enteric bacteria, it serves as a model for studies of endophytic, plant-bacterial associations due to its efficient colonization of plant tissues (including maize and wheat, two of the most important crops in the world), while maintaining a mutualistic relationship that encompasses supplying organic nitrogen to the host plant. Genomic analysis examined *K. pneumoniae* 342 for the presence of previously identified genes from other bacteria involved in colonization of, or growth in, plants. From this set, approximately one-third were identified in *K. pneumoniae* 342, suggesting additional factors most likely contribute to its endophytic lifestyle. Comparative genome analyses were used to provide new insights into this question. Results included the identification of metabolic pathways and other features devoted to processing plant-derived cellulosic and aromatic compounds, and a robust complement of transport genes (15.4%), one of the highest percentages in bacterial genomes sequenced. Although virulence and antibiotic resistance genes were predicted, experiments conducted using mouse models showed pathogenicity to be attenuated in this strain. Comparative genomic analyses with the presumed human pathogen *K. pneumoniae* MGH78578 revealed that MGH78578 apparently cannot fix nitrogen, and the distribution of genes essential to surface attachment, secretion, transport, and regulation and signaling varied between each genome, which may indicate critical divergences between the strains that influence their preferred host ranges and lifestyles (endophytic plant associations for *K. pneumoniae* 342 and presumably human pathogenesis for MGH78578). Little genome information is available concerning endophytic bacteria. The *K. pneumoniae* 342 genome will drive new research into this less-understood, but important category of bacterial-plant host relationships, which could ultimately enhance growth and nutrition of important agricultural crops and development of plant-derived products and biofuels.

## Author Summary

Bacterial endophytes are capable of inhabiting the living tissues of plants without causing them significant harm. *Klebsiella pneumoniae* 342 (Kp342) is a model for this plant host-bacterial association, in part due to its capacity to colonize in high numbers the interior of plants including wheat and maize, two of the most important crops in the world. Kp342 possesses the ability to capture atmospheric nitrogen gas and turn it into an organic form (a process known as nitrogen fixation), of which part may be used as fertilizer by its plant host. Here, we describe the genome sequence and analysis of this model endophyte. When the Kp342 genome is compared to the genome of a closely related pathogenic relative, we can begin to surmise that its preference to engage in a harmonious relationship with plants is a result of many interacting factors. These include differences in its protein secretion systems, the manner in which its genes are regulated, and its ability to sense and respond to its environment. The study of endophytes is increasing in intensity due to the roles they may play in multiple biotechnological applications, including enhancing crop growth and nutrition, bioremediation, and development of plant-derived products and biofuels.

## Introduction

*Klebsiella pneumoniae* 342 (hereafter Kp342) is a mutualistic, diazotrophic (nitrogen-fixing) endophyte and as such is capable of providing small but critical amounts of fixed nitrogen in the form of ammonia by the colonization of the interior of their plant hosts while receiving vital nutrients and protection without inducing symbiotic structures or causing disease symptoms. This form of plant-bacterial association contrasts with other, better studied bacterial interactions with plants in which bacteria can cause disease (pathogens), form obligate associations beneficial to the bacterium which may or may not benefit the plant (symbionts) or colonize the surface of plant structures (epiphytes) [1].

The genus, *Klebsiella*, named after the microbiologist Edwin Klebs, are characterized as rod-shaped, Gram-negative γ-proteobacteria that can live in water, soil, and plants and are pathogenic to humans and animals [2]. In plants, *K. pneumoniae* strains capable of living as endophytes are of interest as they can increase plant growth under agricultural conditions [3], and provide fixed nitrogen to certain grasses [4]–[6]. Culture independent analyses have also suggested the presence of *Klebsiella* in sweet potato [7] and strains have been isolated from the interior of rice [8], maize [9], sugarcane [10], and banana [11]. *Klebsiella* strains may also be human pathogens contaminating the food supply. In humans, certain strains of *K. pneumoniae* are known to cause nosocomial urinary tract infections, and pneumonia, leading to septicemia and death.

Enteric bacteria are frequent inhabitants of the plant interior and can induce plant defenses, thereby reducing their numbers in plants. In particular, strains of *Klebsiella* are routinely found within a variety of host plants [11]–[13]. Flagella are known to induce plant defense [14]–[16]. As *Klebsiella* lack flagella, their high numbers in plants may be attributed at least in part to their lack of extracellular structures that induce plant defenses [17].

Kp342 was isolated from the interior of nitrogen-efficient maize plants [18] as part of a search for nitrogen-fixing endophytes in maize that may be used in the future to reduce the amount of nitrogen fertilizers required for optimum yield. Later work showed that this strain could provide a small amount of fixed nitrogen to wheat under greenhouse conditions [6]. In addition, this strain was found to colonize the interior of a wide variety of host plants with a very small inoculum dose [19]. Kp342 also colonizes the interior of alfalfa sprout seedlings in much higher numbers than other enteric bacteria tested [20].

Plants express two types of defense systems in response to microorganisms in the environment. Systemic acquired resistance (SAR) is induced by plant pathogens and can be stimulated in plants by addition of salicylic acid. Induced systemic resistance (ISR) is induced by bacteria in the rhizosphere and is regulated within the plant by levels of the plant hormones, jasmonic acid and ethylene. Kp342 induces ISR but not SAR while other enteric bacteria induce both systems [17]. Though the molecular basis for nitrogen fixation in *K. pneumoniae* has been well characterized [21], little is known about how plant-associated *K. pneumoniae* isolates promote plant growth without eliciting plant defense mechanisms. Likewise, the potential for endophytic *K. pneumoniae* isolates to cause human disease is also poorly understood and the potential of plant-associated *Klebsiella* strains to act as reservoirs for drug resistance genes is also unknown.

This study presents the whole genome sequence of Kp342 as well as comparative genomic analyses to other sequenced enteric genomes. The Kp342 genome revealed genes for multiple drug resistances as well as genes for virulence to animals, which further motivated experimental verification of antibiotic resistances and infection in mice. The genomic analyses in this study also include a comparison to a closely related clinical strain isolated from sputum [22], *K. pneumoniae* MGH78578 (hereafter MGH78578). In one previous study, MGH78578 was determined to have a limited ability to colonize the interior of wheat roots in comparison to Kp342 [12]; however, its ability to interact with other plants or form other types of plant associations is at present unknown.

The whole genome analyses presented here were completed in order to identify new insights into genetic characteristics that may be influential to the ability of Kp342 to adopt an efficient endophytic lifestyle. Further, these analyses revealed new insights into antibiotic resistance mechanisms, metabolism, surface attachments, secretion systems, and insertion element and transporter content.

## Results

### Genome Features

**Figure 1:**
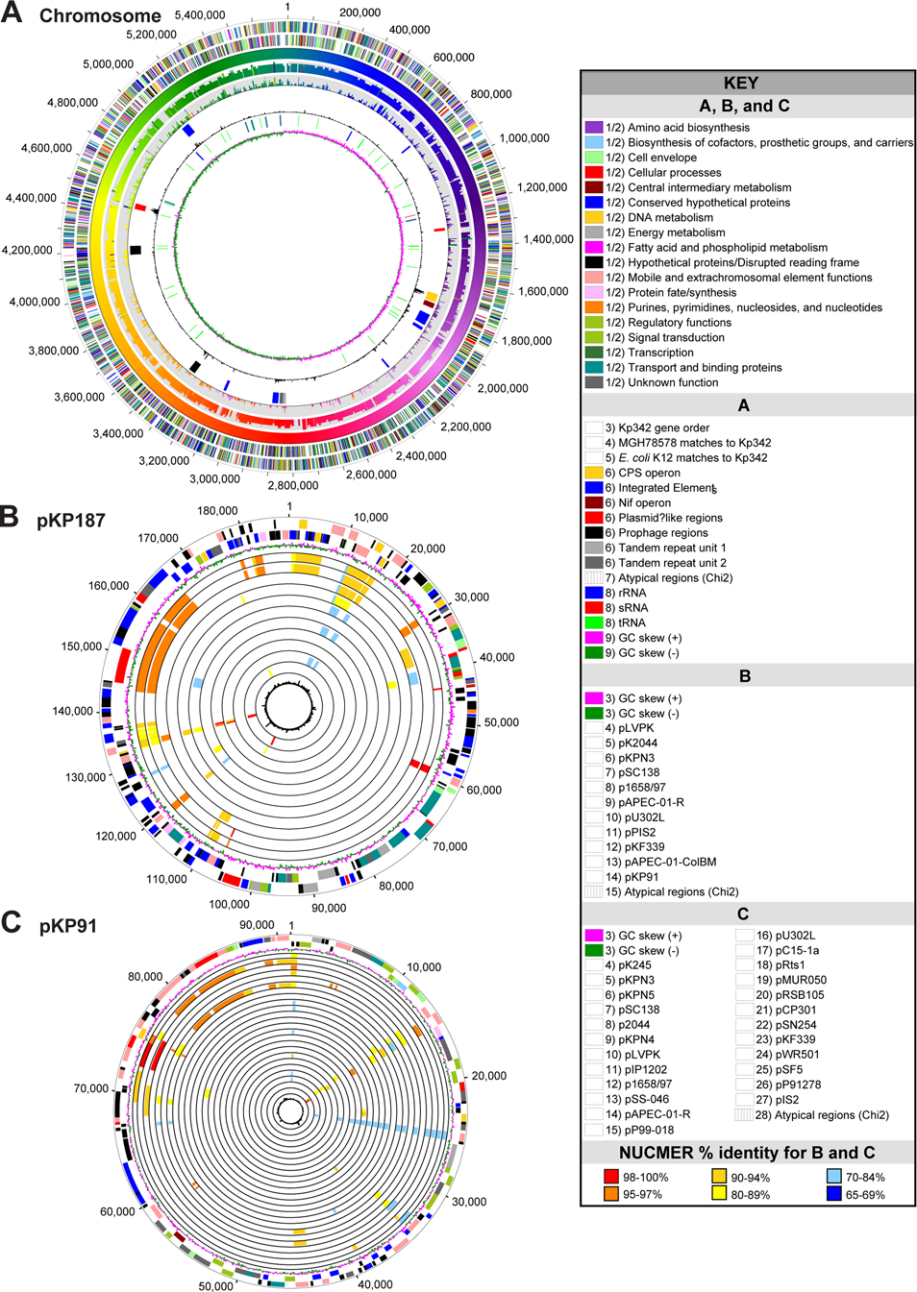
Circular Representation of the Closed Genome of Kp342. The chromosome (A) is illustrated as a circle where each concentric circle represents genomic data and is numbered from the outermost to the innermost circle. Refer to the key for details on color representations and circle number. The comparisons to *E. coli* K12 (circle 5) and MGH78578 (circle 4) are noted as follows. The color indicates the position of the matching Kp342 region (circle 2) using NUCMER. The height of the tick indicates the percent identity of the NUCMER match. Plasmids pKP187 (B) and pKP91 (C) are likewise depicted circular, but each concentric circle from 4 to the innermost circle shows the NUCMER match to previously sequenced plasmids from NCBI, colored by the percent identity of the matching region. See key for color conversion.

The genome of Kp342 is composed of a single circular chromosome of 5,641,239 bp with an overall G+C content of 57.29% (Figure 1) and two plasmids: pKP187, 187,922 bp, 47.15% G+C (Figure 1B); and pKP91, 91,096 bp, 51.09% G+C (Figure 1C). There are eight sets of 5S, 16S and 23S rRNA genes and three structural RNA genes which include 1 tmRNA, 1 SRP/4.5S RNA, and 1 RNAaseP RNA. A total of 88 tRNA genes with specificities for all 20 amino acids and a single tRNA for selenocysteine were identified. The chromosome encodes 5425 putative coding sequences (CDS) representing 88.2% coding density and plasmids pKP91 and pKP187 each encode 113 and 230 putative CDSs having 84.8% and 80.1% coding density, respectively. The preliminary analysis of the genome suggests that of the 5768 total CDSs, 3963 (68.7%) can be assigned biological role categories, while 581 (10.1%) have been annotated as enzymes of unknown function. Conserved hypothetical proteins are represented by 693 (12.0%) CDSs and 531 (9.2%) are hypothetical proteins (Table 1). The average chromosomal gene length is found to be 912 nucleotides, while the average gene length for pKP91 and pKP187 are 638 and 607 nucleotides, respectively. The start codon ATG is preferred (87.9% of the time), while GTG and TTG are used 8.7% and 3.4% of the time, respectively.

**Table 1:** Genome Features of *Klebsiella pneumoniae* 342.

Trait	Chromosome	pKP91	pKP187	Combined
ST^a^	146	–	–	–
MLST allelic profile^a^ *(rpoB:gapA:mdh:pgI:phoE:infB:tonB)*	22:16:30:27:36:24:55	–	–	–
size (bp)	5,641,239	91,096	187,922	5,920,257
G+C content	57.29%	51.09%	47.15%	–
ORF numbers (less pseudogenes)	5,425	113	230	5,768
Pseudogenes	29	8	18	55
Assigned function (less pseudogenes)	3844	49	70	3,963
Amino acid biosynthesis^b^	126	0	0	126
Biosynthesis of cofactors, prosthetic groups, and carriers^b^	202	0	0	202
Cell envelope^b^	419	4	4	427
Cellular processes^b^	319	4	17	340
Central intermediary metabolism^b^	152	1	0	153
DNA metabolism^b^	167	2	6	175
Energy metabolism^b^	691	2	4	697
Fatty acid and phospholipid metabolism^b^	73	0	0	73
Mobile and extrachromosomal element functions^b^	47	20	15	82
Protein fate^b^	235	1	4	240
Protein synthesis^b^	169	0	0	169
Purines, pyrimidines, nucleosides, and nucleotides^b^	91	0	1	92
Regulatory functions^b^	560	13	7	580
Signal transduction^b^	179	2	4	185
Transcription^b^	66	0	1	67
Transport and binding proteins^b^	948	5	15	968
Conserved Hypothetical (less pseudogenes)	625	15	53	693
Unknown function (less pseudogenes)	555	14	12	581
Hypothetical (less pseudogenes)	401	35	95	531
Integrated elements (less phage, IS)	12	0	0	12
Phage regions^c^	2	0	0	2
IS transposase families				
IS3	1	1	3	5
IS5	5	1	1	7
IS6	0	1	0	1
IS110	1	3	0	4
IS481	0	1	0	1
ISL3	0	0	1	1
CRISPRS^d^	0	0	0	0
Protein secretion systems				
Chaperone/usher pathway (fimbriae)	9	0	0	9
Lol	1	0	0	1
Sec	1	0	0	1
Single accessory/two-partner	2	0	0	2
SRP	1	0	0	1
TAT	1	0	0	1
Type I	1	0	1	2
Type II	1	0	0	1
Type III	0	0	0	0
Type IV	1	0	0	1
Type V (autotransporter)	0	0	1	1
Type VI	3	0	0	3
Bacterial adherence				
Type IV pili/other conjugal systems	2	0	0	2
Fimbriael systems	10	0	0	10
FN-binding proteins^g^	0	0	0	0
Motility e	1	0	0	1
Two-component systems^e^,^f^				
Response regulator (PF00072)	40	0	0	40
Sensor histidine kinase (PF02518)^g^	31	0	0	31
Toxin production and resistance^e^	100	1	13	114
Transporters				
Total proteins	867	6	15	888
Number per Mbp	154	66	80	299
ABC Family	417	0	5	422
MFS Family	125	2	1	128
2-HCT Family	3	0	0	3
DASS Family	8	0	0	8

a ST and MLST allelic profiles follow the PubMLST Web site (http://pubmlst.org/).

b An ORF can be assigned multiple main role categories.

c Putative prophage regions predicted by PhageFinder (50).

d Putative CRISPR region predicted by CRISPRFinder (27).

e Based on TIGR role category.

f Based on HMM results.

g Less topoisomerases, MutL and Hsp90.

The larger of the two plasmids, pKP187, is most similar to the *K. pneumoniae* CG43 virulence plasmid pLVPK [23] at the nucleotide level (Figure 1B). Use of the genome alignment program, NUCMER [24], revealed that the similarity is mainly limited to regions of the plasmid encoding replication, partitioning/maintenance, arsenate and tellurite resistance, and transposase/recombinase functions. Unlike pLVPK, which has only one, pKP187 encodes two replication genes, which are 46% identical at the protein level and both are recognized by PF01051, Initiator Replication protein. The first *rep* gene (KPK_A0248) was chosen as the origin of replication because it is flanked by iteron repeat sequences. The second *rep* gene, KPK_A0025, did not have detectable flanking iteron repeat structures, but was most similar to *repA* of pLVPK. Another notable difference between pLVPK and pKP187 is the absence from pKP187 of the virulence-associated iron-acquisition siderophore systems and CPS biosynthesis control loci *rmpA* and *rmpA2*. This plasmid (pKP187) also encodes a putative innate immunity cationic antimicrobial peptide resistance protein, PagP (formerly CrcA) (KPK_A0097) [25].

The smaller plasmid, pKP91 also has two *rep* genes, *repA* (KPK_B0121) and *repE* (KPK_B0094) and has the most overall nucleotide similarity to *K. pneumoniae* plasmids pK245, pKPN3, and pKPN4 (Figure 1C). This similarity is restricted to regions of the plasmids conferring replication, partitioning, conjugal transfer, and transposon functions. The origin of replication was chosen downstream of *repA*, which has 95% protein identity to *repA* of the IncFII *K. pneumoniae* plasmid pGSH500, so that nucleotide one of the DnaA box (TTATTCACA) is the beginning of the plasmid sequence [26]. This plasmid also encodes a plasmid addiction module (KPK_B0088 and KPK_B0087), as well as several oxidoreductase genes, and a putative fusaric acid resistance gene.

Full-length transposase genes were manually annotated with the assistance of the ISFinder database (http://www-is.biotoul.fr/). Twenty full-length and 17 fragmented insertion sequence (IS) elements, belonging to six transposase families were identified in the Kp342 chromosome and two plasmids. These IS elements encoded four different IS3 transposases, one IS5 transposase, one IS6 transposase, three different IS110 transposases, one IS481 transposase, and one ISL3 transposase. Most of the IS elements are segregated to either the chromosome or one of the plasmids. However, the seven copies of the IS5 family element, which are 99% identical at the protein level to IS903B in the database, have been identified in all three DNA molecules with five copies in the chromosome and one copy in each of the plasmids. Therefore, it is likely that the chromosome and two plasmids have been in close association long enough for dissemination of IS903B from one DNA molecule to the other two. Also, measuring the number of full-length IS elements in each kb of the three DNA molecules reveals approximately 20- to 60-fold higher density of insertions in the plasmids compared to the chromosome with seven copies in the ∼5641 kb chromosome, five copies in ∼187 kb of pKP187, and seven copies in ∼91 kb of pKP91.

The genome was examined for the presence or absence of clustered regularly interspaced short palindromic repeats (CRISPRs) using CRISPRFinder [27]. No functional CRISPR system was determined in Kp342 or MGH78578 although they have been identified in other closely related enteric bacteria including all genomes of the genera, *Escherichia* and *Salmonella* sequenced to date. Recently CRISPRs have been linked to the acquisition of resistance against bacteriophages [28],[29].

### Overview of Metabolism in Kp342

Analyses of the Kp342 genome reflected its most distinguishing features as a diazotroph, facultative anaerobe and an endophyte. Genome analyses confirmed each of these abilities while also revealing fundamentally new insights into the metabolic potential of this organism. Of particular importance was the presence of a large complement of genes devoted to carbohydrate, including cellulosic and aromatic compound degradation, many of plant origin. These traits are likely to make Kp342 important to carbon and nutrient cycling and its ability to form endophytic associations. However, this gene complement may also prove useful for further exploration in biotechnological applications including conversion of cellulose to biofuels and the bioremediation of aromatic compounds. For a general synopsis of central intermediary and energy metabolism, including sulfur and phosphorous metabolism, and electron transport, refer to Text S1. Highlights of the nitrogen cycle, sugar, cellulosic and aromatic metabolism in Kp342 are described below.

### The Nitrogen Cycle

Among the fundamental roles that Kp342 plays in the nitrogen cycle is its capacity to fix nitrogen [6],[18], which was confirmed through genome analyses by the presence of a nitrogen fixation regulon (KPK_1696-KPK_1715) (Figure 1A; Figure S1). In contrast, comparative genomic analyses determined that genes associated with nitrogen fixation including nitrogenase, the enzyme central to this process, are absent in MGH78578. It is therefore presumed that MGH78578 cannot fix nitrogen. Central reactions of the nitrogen cycle which Kp342 can perform based on genome analyses are the uptake of nitrate using an assimilatory nitrate and nitrite reductase, respectively (KPK_2087-KPK_2086) and use of nitrate as a terminal electron acceptor in the absence of oxygen.

Of further importance to its role in the nitrogen cycle is the ability of Kp342 to degrade urea to ammonia and carbon dioxide via both the urease complex (which is present in MGH78578) and the two-step reaction catalyzed by urea amidolyase [30] (KPK_2626-KPK_2627) which is absent from MGH78578. The ability to serve additional roles within the nitrogen cycle was also revealed. For example, the presence of a nitrile hydratase (KPK_2673-KPK_2672) which catabolizes various nitrile compounds to their corresponding amides is a feature not noted in other enteric genomes sequenced to date including MGH78578.

### Carbohydrate Metabolism

#### Cellulosic Metabolism

Cellulose is the most abundant carbohydrate in the biosphere followed by starch of which both are widely produced by plants [31]. The association of Kp342 with plants is greatly suggested by the wide variety of genes devoted to the transport and metabolism of these compounds. Of particular importance was the elucidation of a gene complement capable of hydrolyzing α-linked glucans of starches and pectins and another capable of splitting 1,4-β-glucosidic bonds of cellulosic components and long chain polymers of beta-glucose such as chitin. At least 38 genes were placed into 16 glycosyl hydrolase families that could be assigned functions belonging to O-glycosyl hydrolases (EC 3.2.1-) responsible for the hydrolysis of glycosidic bonds between two or more carbohydrates, or between a carbohydrate and a non-carbohydrate compound [32]. Of these, 35 were found on the main chromosome and three on the plasmid, pKP187.

At least two genes can be confidently assigned functions (and EC numbers) related to the decomposition of highly ordered forms of insoluble cellulose [33], KPK_A0121, cellulose 1,4-beta-cellobiosidase (*celK*) (EC#3.2.1.91), an exoglucanase and KPK_0224, cellulase (*bcsZ*), (EC#3.2.1.4), an endogluconase. Additional genes encoding enzymes with specificity towards 1,4-β-glucosidic bonds and most likely act by hydrolyzing short cello-oligosaccharides include: KPK_2587, beta-glucosidase (*bglH*), (EC#3.2.1.21), a cytoplasmic beta-glucosidase, and KPK_1599, beta-glucosidase (*bglX*), (EC#3.2.1.21), its periplasmic form.

#### Cellulosic Metabolism–Plasmid Associations

Of the three glycosyl hydrolase genes found on pKP187, two were co-localized, the aforementioned KPK_A0121 and a putative glucan 1,4-beta-glucosidase (*celD*) (KPK_A0120), whose probable function is involved in sequentially cleaving 1,4-beta-D-glucosidic linkages from the non-reducing end of crystalline cellulose or cello-oligosaccharides. An additional member of the glycosyl hydrolase 1 family was also found (KPK_A0131). As a probable cellobiase the gene product is also likely responsible for the hydrolysis of terminal, non-reducing beta-D-glucose residues with release of beta-D-glucose.

Phylogenetic analyses of the predicted protein sequences of the *celD* (Figure S2A) and *cel*K (Figure S2B) homologs revealed that they are more closely related to non-enteric bacteria. For example, the closest relatives to the *celD* homolog are *Vibrio shiloni* and *Photobacterium* sp. SKA34, which are marine dwelling γ-proteobacteria. In the case of the *celK* homolog, the closest relatives are to the low G+C firmicutes including members of the genus, *Clostridium*. The determination of these genes on a plasmid along with the results of the phylogenetic analyses including the lack of homologs in MGH78578 suggests that their presence in the Kp342 genome could be the result of a lateral transfer event although other mechanisms such as gene loss, or even sampling bias could be responsible for the incongruent results of the phylogenetic gene trees when compared to 16S rRNA-based trees.

#### Conversion of Hemicellulosic Substrates to Sugars

Genome analyses also revealed an ability to convert various hemicellulosic substrates to fermentable sugars. For example, the Kp342 genome possesses the ability to metabolize common components of xylan, arabinose and xylose. Genes related to this metabolism include duplications of *xylA* (xylose isomerase) (KPK_0176, KPK_4922) and *xylB* (xylulokinase) (KPK_0177, KPK_1623) responsible for creating the phosphorylated derivative, D-xylulose 5-phosphate. The genome also possesses beta-1,4-xylosidase (KPK_4924) responsible for the hydrolysis of 1,4-beta-D-xylans and alpha-N-arabinofuranosidase (KPK_4626). Arabinofuranosidases work synergistically with xylanases to degrade xylan to its component sugars.

In addition to synthesis of glycogen, the Kp342 genome also encodes genes capable of degrading the α-linked glucans (primarily 1,4-α and 1,6 α-linkages) of glycogen, plant starches and pectins as well as the degradation of low molecular weight carbohydrates produced from their breakdown such as maltodextrins, pullulan and D-galacturonate. Genome analyses also revealed the ability to metabolize a wide variety of five and six carbon sugars including, fructose, fucose, rhamnose, arabinose, galactose and glucose and sugar alcohols such as mannitol (to fructose) and sorbitol (to fructose).

### Aromatic Compound Degradation via Oxidation and Decarboxylation

Aromatic compounds are abundantly distributed throughout the environment [34]. A frequent source of these compounds in nature is the result of the breakdown of lignin from plants [35] as well as the result of anthropogenic inputs. As compounds often present in plant cells, these molecules can act as signals for bacteria when in close proximity to the plant and may be important influences on plant colonization [1].

Genome analyses identified the potential of Kp342 to oxidatively catabolize a variety of low-molecular mass aromatic compounds, many of which arise from lignin degradation, including ferrulic acid, vanillate (KPK_2715, KPK_2713, KPK_2433 KPK_2298) and 2-chlorobenzoate (KPK_2486-KPK_2484) to the central aromatic ring metabolites, protochatechuate and catechol [36],[37]. Genome analyses further elucidated the presence of a protocatechuate pathway in which ring cleavage is subsequently mediated by the 3,4-protocatechuate dioxygenase (KPK_2400-KPK_2401), and the ortho cleavage pathway of catechol, in which ring cleavage is mediated by catechol 1,2-dioxygenase (KPK_2483) [36],[37]. The Kp342 genome also possesses a complete β-ketoadipate pathway (KPK_2916-KPK_2914) for further degradation of the ring cleavage products to TCA cycle intermediates [36],[37]. Additional ring hydroxylating dioxygenases were identified in the Kp342 genome although their substrate specificities or the pathways in which they participate are less well known. They are described in Text S1.

Genome analyses also revealed that the Kp342 genome may also be capable of reductive, non-oxidative decarboxylations of some aromatic compounds. For instance, the genome possesses CDSs encoding the multi-subunit 4-hydroxybenzoate decarboxylase enzyme capable of decarboxylating 4-hydroxybenzoate to phenol and carbon dioxide (KPK_1027-KPK_1025).

### Small Molecule Transport

Kp342 possesses an exceptionally robust transporter repertoire, encoding 888 transporter genes (15.4%), one of the highest percentages of CDSs functioning as transporters identified to date (Table S1). The total number of transporters is similar to plant/soil-associated microbes, such as *Bradyrhizobium japonicum* (986, 11.9%), *Mesorhizobium loti* (885, 12.2%) and *Agrobacterium tumefaciens* (835, 15.5%) [38],[39].

The distribution of transporter families is similar to the *Enterobacteriaceae*; however, Kp342 exhibits an expansion in the majority of transporter families analyzed. For example, the genome encodes 422 (7.3%) ATP-binding cassette (ABC) family transporter genes and 128 (2.2%) Major Facilitator Superfamily (MFS) genes (the highest number of MFS genes in all sequenced prokaryotic genomes) while *Escherichia coli* K12 encodes 210 (5.0%) and 70 (1.7%) genes respectively. Transporters in these families are involved in the uptake of various nutrients, such as sugars, amino acids, peptides, nucleosides and various ions, as well as the extrusion of metabolite waste, toxic byproducts and antibiotics.

There are also several families of transporters present in *K. pneumoniae* but absent in *E. coli*, including the *citW* (KPK_4687), *citS* (KPK_4716) and *citX* (KPK_4686) homologs of the 2-hydroxycarboxylate transporter (2-HCT) family. Many species of enterobacteria, including *K. pneumoniae* and *E. coli* can grow with citrate as the sole carbon and energy source [40]. Transporters in the 2-HCT family are responsible for the uptake of citrate. CitW transports H^+^ and citrate in exchange for acetate, the product of citrate fermentation, and is expressed only under anoxic conditions where acetate is the main end-product of citrate fermentation [41]. CitS and KPK_1918 are sodium ion-dependent citrate permeases [42]. CitX facilitates transfer of the prosthetic group (2′-(5″-triphosphoribosyl)-3′-dephospho-CoA) to the citrate lyase gamma chain. In contrast, *E. coli* K12 encodes a single protein, CitT, a Divalent Anion:Sodium Symporter (DASS) family transporter, for the uptake of citrate. Kp342 encodes additional transporter families for the uptake and efflux of Ni^2+^, Co^2+^ Zn^2+^, Fe^2+^ and Mg^2+^ that are absent in *E. coli* K12, including 3 members of the Ni^2+^-Co^2+^ Transporter (NiCoT) Family, 1 member of the Zinc (Zn^2+^)-Iron (Fe^2+^) Permease (ZIP) Family, and 2 members of The Mg^2+^ Transporter-E (MgtE) Family. When compared to Kp342, the clinical strain MGH78578 encodes slightly fewer transporter genes, 836 transporter genes (16.1% of CDSs). Although the transporter family distribution is nearly identical to Kp342, a lesser degree of expansion in ABC and MFS transporter families was noted in the clinical strain.

### Protein Secretion Systems

The genome of Kp342 encodes ten of eleven known protein secretion systems (Table 1). The only protein secretion system not found in the genome is the Type III or contact-dependent protein secretion system, which is commonly used by plant and animal pathogens to secrete effector proteins into the cytoplasm of eukaryotic cells [43]. Kp342 possesses the Sec-dependent and Sec-independent (twin-arginine translocation “TAT”) protein export pathways for the secretion of proteins across the inner/periplasmic membrane. In addition, genome analyses identified that Kp342 possesses the signal recognition particle (SRP) and two-partner secretion (TPS)/single accessory pathway, *lol*, Type I, Type II, Type IV, Type V or autotransporter, and Type VI secretion systems. The Type II secretion system in Kp342 is essentially identical to the prototypical Type II secretion pathway that was first discovered in *K. pneumoniae* UNF5023 for the secretion of pullulanase, a starch debranching lipoprotein [44]. The Type IV secretion system is present on integrated element IE04 and may be part of a conjugal transfer system. The Type VI secretion system was recently discovered in *Vibrio cholerae* for the secretion of virulence factors encoded by *hcp* and *vgr* loci [45].

The chaperone/usher pathway is a major terminal branch of the sec pathway used to translocate fimbrial components across the Gram-negative outer membrane [46]. A large number of chaperone/usher pathway units were identified in both the Kp342 (9) and MGH78578 (11) genomes as determined by HMM scores above the trusted cut off to PF00577, Fimbrial Usher protein (Figure S3). This was significantly more in comparison to multiple strains of other plant pathogenic genera (1 per *Erwinia*, *Agrobacterium*, *Xanthomonas*, and *Xylella* genome, and 2.2 per *Pseudomonas* genome) (Figure S3). Similarly, the average number of PF00577 matches to multiple strains of the marine pathogenic *Vibrio* and *Aeromonas* genera was 1 or less per genome. In contrast, many of the enteric pathogenic genera, *Escherichia*, *Salmonella*, *Shigella*, and *Yersinia*, have more than 8 chaperone/usher units per. The genome of *Photorhabdus luminescens*, an enteric mutualist and insect pathogen, has 8 chaperone-usher units.

### Site-Specific Integrated Elements and Bacteriophages

A total of thirteen site-specific integrated elements have been identified in the genome of Kp342, including two putatively integrated plasmids and two prophages. The data compiled for these integrated elements is presented in (Table S2). Twelve of the thirteen site-specific recombinases were from the tyrosine recombinase family and targeted either tRNAs or inserted in tandem into tRNA-derived sequences (8), genes (3) or intergenic regions (1). Where possible, putative element boundaries were determined by locating flanking direct repeats, indicative of the core attachment sequence. Many of these repeat-flanked regions were confirmed by other data such as insertion within an operon or by atypical G+C%.

IE01 appears to be a phage-like bacteriocin, analogous to *Pseudomonas pyocins*, which encodes phage tail fibers and lytic enzymes, with a nested insertion into the 5′ end of *umuC* by another element IE01b. IE02 encodes a beta-ketoadipyl CoA thiolase (KPK_1840), an MFS-family transporter (KPK_1839), and a polyketide synthase (KPK_1838) that may be used by Kp342 to convert plant-derived aromatic compounds to acetyl-CoA and succinyl-CoA and subsequently into a polyketide, which may be expelled from the cell by a CDS having high sequence similarity to a methylenomycin A resistance efflux pump (KPK_1835). It is interesting that KPK_1841- KPK_1838 protein sequences have high identity and synteny to *Chromobacterium violaceum* ATCC 12472 genes CV4290-CV4293 and KPK_1836- KPK_1835 with CV0720-CV0719, suggesting that these genes may exist as mobile functional units. IE03 encodes three proteins, which may be involved in the synthesis of putrescine and metabolism of polyamines. IE04 encodes a type IV secretion system (KPK_1774- KPK_1789). These protein sequences have best BLASTP matches to the *Erwinia caratovora* subsp. *atroseptica* plasmid-like integrated element HAI7 (ECA1612-ECA1627) [47]. Though this secretion system may very well be involved in conjugal transfer of DNA, it may also have a dual role in the secretion of virulence determinants, as was shown in *E. caratovora* [47]. Analyses of IE05, IE07 and IE10 revealed the presence of tyrosine recombinases, while all other CDSs identified encode only proteins with unknown function. IE06 encodes a type I restriction-modification system as well as two acetyltransferase genes, a putative glyoxalase, and a glyceraldehyde-3-phosphate dehydrogenase. It is unclear if any of these enzymes would have a selective advantage; however, this integrated element encodes a protein (KPK_4954) with similarity (37.8% identity and 57% similarity over 2782 aa) to NdvB of *Rhizobium meliloti*, a protein required for the synthesis of cyclic Beta-(1,2)-glucan, nodule invasion and bacteroid development [48], possibly having a role in osmotic adaptation [49]. IE08 and IE09 appear to be integrated plasmids, encoding genes with similarity to plasmid replication genes, partitioning genes and mobilization genes, but carry no genes with identifiable function. Similar to IE11, IE01, encodes proteins homologous to UmuC and UmuD; however, unlike IE01, IE11 also encodes RecE and RecT DNA repair enzymes.

In addition to the 11 site-specific integrated elements described above, the genome of Kp342 also harbors 2 prophage genomes. Both prophage regions were predicted by Phage_Finder [50]. PHAGE01 is predicted to be 36346 bp in size, with a G+C% of 47.4%, and appears to have inserted into KPK_3407 (isocitrate dehydrogenase) at nucleotide positions 3425830-3389485 (Table S2). PHAGE02 is slightly larger (48557 bp) with a slightly higher G+C content of 52.8%. It is inserted into a tRNA-Arg at nucleotide coordinates 4230390-4181834. Both regions and all integrated elements had G+C% compositions less than the whole Kp342 chromosome (57.3% G+C). PHAGE01 has 7 out of 22 possible best matches (using Phage_Finder) to *Klebsiella* phage while PHAGE02 has 7 out of 44 possible best matches to *Xanthomonas* phage OP2.

### Comparative Genome Analysis

#### Kp342 and MGH78578

**Figure 2:**
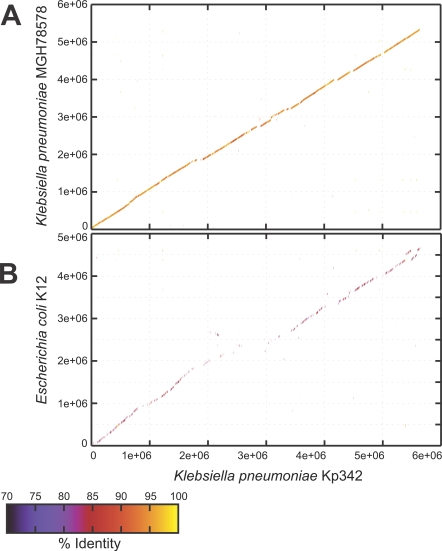
Whole-Genome Comparison of Kp342 to *K. pneumoniae* MGH78578 and *E. coli* K12. Line figures depict the results of NUCMER analysis. Colored lines denote nucleotide percent identity and are plotted according to the location in the reference Kp342 genome (x-axis) and the query genomes *K. pneumoniae* MGH78578 (A) and *E. coli* K12 (B).

The genomic structure of Kp342 was highly syntenic when compared to the genome of the recently sequenced clinical isolate MGH78578 (Figure 2A) with an average nucleotide identity of 95% over 4822472 Kp342 nucleotides. Many of the breakpoints in synteny correspond to the presence or absence of integrated elements and prophages. This conserved gene order was not limited to the *Klebsiella*, but can be expanded to *E. coli* K12 (Figure 2B), with an average nucleotide identity of 85% over 1146557 Kp342 nucleotides.

**Figure 3:**
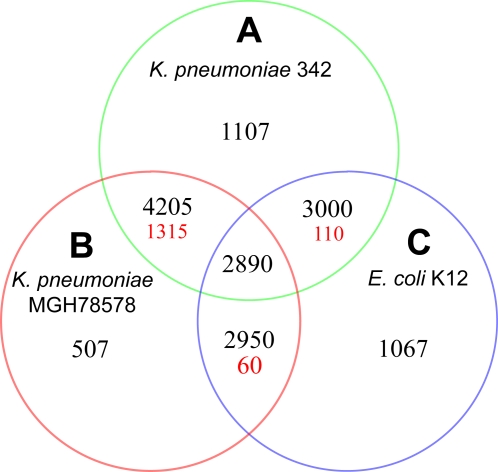
Whole Genome Comparison of *K. pneumoniae* 342, *K. pneumoniae* MGH78578, and *E. coli* K12 Proteins. The Venn diagram shows the number of proteins shared (black) or unique (red) within a particular relationship for all three organisms compared.

A comparative study was undertaken to determine putative orthology between the Kp342, MGH78578 and *E. coli* K12 genomes (Figure 3, Tables S3, S4, S5 and S6). These results revealed 4205 putative orthologs were shared between Kp342 and MGH78578 with an average protein percent identity of 96% (Table S3). When this 4205 member protein set was further analyzed for identification of the fraction not found in *E. coli* K12 (and thus specific to *Klebsiella*) 1315 putative orthologs were determined (Figure 3, Table S4). A total of 1107 genes were identified as exclusive to Kp342 (not in MGH78578 or *E. coli* K12) (Figure 3, Table S5) and 507 were exclusive to MGH78578 (Figure 3, Table S6). In contrast only 110 putative orthologs were shared between Kp342 and *E. coli* K12 (not present in MGH78578) (Figure 3, Table S7) and 60 shared between MGH78578 and *E. coli* K12 (not in Kp342) (Figure 3, Table S8).

From this study several important differences between the Kp342 and MGH78578 genomes are evident which may have important implications concerning their preferred lifestyle and host range (endophyte for Kp342 and human pathogen presumably for MGH78578). A clear difference is present in transcription factor content and signaling proteins which may contribute to dissimilarities in the regulatory networks of these two organisms. The Kp342 genome possesses forty-eight transcription factors classified in at least nine families of transcriptional regulators of diverse function and five additional CDSs annotated as putative transcription factors not found in MGH78578 (Table S5). Conversely, six transcription factors from three transcription factor families (LysR, DeoR, IclR) were identified in MGH78578 but not Kp342 (Table S6). In addition, at least two anti-anti-sigma factors (KPK_3076 and KPK_3564) are present in Kp342 which are not found in MGH78578 (Table S5). Anti-anti-sigma factors play critical roles in regulating the expression of alternative sigma factors in response to specific stress signals [51]. The anti-anti-sigma factors identified here each posses a Sulfate Transporter and AntiSigma factor antagonist (STAS) domain and are paralogs of one another. Therefore, they are presumably related by gene duplication, but they may have different physiological functions that remain to be determined in Kp342.

At least 13 genes whose functions are related to signal transduction in Kp342 were not identified in MGH78578 (Table S5). These include members of two-component systems (KPK_2666, KPK_3077, KPK_3085), the phosphotransferase system important to active transport and regulation of carbohydrate uptake, and regulators of the global secondary messenger protein cyclic diguanylic acid (c-di-GMP), specifically diguanylate cyclases and c-di-GMP phosphodiesterases (KPK_2890, KPK_3355, KPK_3356, KPK_3392, KPK_3558, KPK_3794).

Bacterial surface-associated structures such as fimbriae have been determined to play a role in bacterial adhesion to host cells including plants and animals and in biofilm formation [1],[52]. Several differences in fimbrial content were noted between the two strains. The Kp342 genome contains three fimbrial proteins (KPK_0824, KPK_2632 and KPK_2633) not present in MGH78578 (Table S5). Conversely MGH78578 possesses at least 13 CDSs annotated as structural proteins, or members of a chaperon/usher system not found in Kp342 (Table S6). This set includes homologs to the *stb* fimbrial operon of the human pathogen *Salmonella enterica* serotype Typhimurium, which was reported to be critical to persistence of this organism in the gut of mice [52].

Differences in the distribution of genes devoted to Type IV and Type VI secretion systems were noted in this study between Kp342 and MGH78578. The Type IV secretion system identified on integrated element IE04 in Kp342 is absent in MGH78578 as well as an additional Type IV pilus assembly family protein (KPK_0839) (Table S5). The Kp342 and MGH78578 genomes appear to share core components of the less well-known TypeVI secretion system [45]. However, at least four CDSs determined in Kp342 putatively involved in TypeVI secretion, were not found in MGH78578 (KPK_2042, KPK_3066, KPK_2055, KPK_2056) (Table S5).

#### Phytobacteria

Only one other complete genome of an endophyte has been described, *Azoarcus* sp. BH72 [53]. A comparison of the Kp342 genome to BH72 failed to elucidate any CDSs shared uniquely between these genomes. Therefore, to better identify CDSs that are important for a plant-associated lifestyle, protein sequences of Kp342 were compared to those of 28 completely sequenced phytobacteria representing other plant-bacterial relationships (e.g., plant pathogens, epiphytes, and saprophytes). These include the following: *Acidovorax avenae* subsp. *citrulli* AAC00-1, *Agrobacterium tumefaciens* C58, *Bradyrhizobium japonicum* USDA 110, *Burkholderia cenocepacia* AU 1054 and HI2424, *Erwinia carotovora* subsp. *atroseptica* SCRI1043, *Leifsonia xyli* subsp. *xyli* CTCB07, *Mesorhizobium loti* MAFF303099, Onion yellows phytoplasma OY-M, *Pseudomonas aeruginosa* PAO1 and UCBPP-PA14, *Pseudomonas fluorescens* Pf-5 and PfO-1, *Pseudomonas syringae* pv. *phaseolicola* 1448A and pv. *syringae* B728a and pv. *tomato* DC3000, *Ralstonia solanacearum* GMI1000, *Rhizobium etli* CFN 42, *Rhizobium leguminosarum* bv. *viciae* 3841, *Sinorhizobium meliloti* 1021, *Xanthomonas axonopodis* pv. *citri* 306, *Xanthomonas campestris* pv. *campestris* 8004 and ATCC 33913 and pv. *vesicatoria* 85-10, *Xanthomonas oryzae* pv. *oryzae* KACC10331 and MAFF 311018, and *Xylella fastidiosa* 9a5c and Temecula1.

A total of 45 proteins fell into this “phytobacteria only” bin (Table S9). The top three main functional biological role categories were: Hypothetical proteins or proteins of unknown function (17), Transport and binding proteins (9), and Central intermediary metabolism (5). Although the ability of MGH78578 to form plant-associations is not well known given that it is a clinical isolate if this genome were considered in this analysis as part of the non-phytobacteria (and therefore a phytobacterial-only gene cannot have a match in the MGH78578 genome) this bin decreased to 23. The top three main functional biological role categories were: Hypothetical proteins or proteins of unknown function (9), Central intermediary metabolism (4) and Energy metabolism (2) and Transport and binding proteins (2).

### Plant-Induced and Associated Genes

Many studies have been conducted on plant-associated bacteria to identify genes that are induced during colonization or growth associated with plants [54]-[60]. These studies used variations on the original *in vivo* expression technology (IVET) [61]. A total of 231 protein sequences that were found to be plant-induced in these studies were used to query the CDS sequences of Kp342 and MGH78578 (Table S10). Of the 231 known plant-induced query sequences searched with WUBLASTP, 75 (32.5%) had significant matches (*p*-value ≤less 10^−5^; identity ≥35%; no alignment length restriction) to Kp342 proteins. These were distributed among 17 different role categories (Table S10). The top five main role categories were Energy metabolism (12.6%), DNA metabolism (10.3%), Regulatory functions (10.3%), Unknown function (9.2%), and Transport and binding proteins (8%). Twelve of the 75 known plant-induced proteins had two or three matches to Kp342 proteins. These include ipx53/hopAN1, ipx59 and 61, Ripx109, 117, 127, 151, 152, 24, 52, 58 and 99 (Table S10). Many of these plant-induced genes are thought to function in colonization and evasion of plant defenses. No known plant effector or avirulence proteins were identified in the genome of Kp342.

Several amino acid and nucleotide biosynthesis genes present in Kp342 were found to be induced in *Ralstonia solanacearum* and *Pseudomonas syringae* pv. *tomato* upon plant colonization. These genes include KPK_0998 (CTP synthase (*pyrG*)), KPK_2276/ KPK_0844 (acetyl-CoA acetyltransferase), KPK_1442 (amidophosphoribosyltransferase (*purF*)), KPK_0542 (argininosuccinate synthase (*argG*)), KPK_0863 (diaminopimelate decarboxylase (*lysA*)), and KPK_4659 (acetolactate synthase large subunit (*ivlI*)) [55],[57]. Putative stress response genes expressed in *R. solanacearum* upon plant colonization presumably in response to plant defenses were also found in Kp342, including KPK_1518 (a regulatory protein of adaptive response, *ada*), KPK_5230 (excinuclease A (*uvrA*)), KPK_5244 (DNA-damage-inducible protein F (*dinF*)), KPK_2941 (fumarate hydratase (*fumC*)), and KPK_4236 (acriflavin resitance protein A (*acrA*)) [57].

A gene believed to be involved in plant attachment has also been identified independent of the plant-inducible gene searches. This plant inducible haemagglutinin gene in *R. solacacearum* (Ripx150, Table S10) is homologous to a Kp342-specific (Table S5) HecA-like filamentous haemagglutinin (KPK_4110) protein [57]. The *hecA* gene is part of a HecA/B hemolysin/hemagglutinin secretion operon. The HecA/B proteins make up a two-partner secretion (TPS) system in which a TpsA family exoprotein with specific conserved secretion signals is transported across the membrane by a TpsB family channel-forming transporter that recognizes the secretion signal [62]. In *Erwinia chrysanthemi*, a mutant in the *hecA* gene that encodes an adhesin had reduced attachment, cell aggregate formation, and virulence on *Nicotinia clevelandii* [63]. Homologs of this gene appear in both plant and animal pathogens [63].

### Survival Against Plant Defenses

Plants use a variety of non-specific tactics to defend against bacterial, viral and fungal threats, which include the production of reactive oxygen species (ROS) (superoxide, hydroperoxyl radical, hydrogen peroxide, and hydroxyl radical species), nitric oxide, and phytoalexins [64],[65]. The genome of Kp342 encodes mechanisms to protect itself from these three plant defense mechanisms. There are three superoxide dismutases, *sodA* (KPK_5462), *sodB* (KPK_2353) and *sodC* (KPK_2364), four putative catalases (KPK_2233, KPK_2536, KPK_3205, and KPK_3339), 6 putative peroxidases, 1 hydroperoxide reductase (encoded by *ahpC*, KPK_3924 and *ahpF*, KPK_3923), and 12 putative glutathione-S-transferase (GST) or GST domain/family proteins (compared to 7 in *E. coli* K12) that can defend the cell against ROS. Additionally, there is an apparent ability to detoxify the free radical nitric oxide as revealed by the presence of CDSs specific for aerobic nitric oxide detoxification (flavohemoprotein, KPK_1245) and the anaerobic nitrate reduction operon (*nor*RVW, KPK_1083, KPK_1081, KPK_1080) [66]. Lastly, it has been recently shown that the RND-family AcrAB (KPK_4236/ KPK_4237) efflux pump is required for the export of apple tree pytoalexins by *Erwinia amylovora* [67].

### Pathogenicity of Kp342

Before the widespread agricultural use of strains such as Kp342 can be considered, the virulence potential of this strain in an animal model required investigation. A comparison of Kp342 with the type strains of *K. pneumoniae* and *K. oxytoca* by DNA:DNA hybridization showed that Kp342 is a strain of *K. pneumoniae* [12]. As many virulence factors in *K. pneumoniae* have been proposed based on attenuation of signature-tagged mutants [68],[69], and IVET [70], the presence or absence of these factors in the Kp342 genome were examined (Table 2; Tables S11, S12 and S13). A total of 133 nucleotide sequences (93 from Lawlor [69] (Table S11), 16 from Struve [68] (Table S12), and 20 from Lai [70] (Table S13)) were searched against the Kp342 and MGH78578 CDSs using WUBLASTN or against the Kp342 and MGH78578 genomes using BLASTX. Only four examples were found where potential virulence factors were present in Kp342, but absent from MGH78578 (Table 2). However, there were 7 examples based on results of the Lawlor study [69] where the clinical isolate MGH78578 had significant matches that were missing from the endophyte Kp342 (Table 2). It is not directly apparent how these mutants affect virulence except for the mutant designated #39-13, which encodes a fimbrial-like protein that may be necessary for attachment to the host.

**Table 2:** Lawlor et al. Signature-tagged Mutants Present in One Strain but Lacking from the Other*.

Strain #	Kp342			MGH78578			Description from genome annotation
	match	%id	*e*-value	match	%id	*e*-value	
99-44	KPK_1773	71	6.20E-52	-	-	-	type IV conjugative transfer system coupling protein TraD
9-35	KPK_5395	97	8.00E-88	-	-	-	undecaprenyl-phosphate á-N-acetylglucosaminyl 1-phosphatetransferase (*wecA*)
8-41	KPK_5049	94	9.40E-40	-	-	-	methionine-S-sulfoxide reductase (*msrA*)
32-14	KPK_3791	91	8.40E-37	-	-	-	conserved hypothetical protein
77-25	-	-	-	gi152969435	98	3.00E-32	*ybjL* hypothetical protein
26-23	-	-	-	gi152969874	97	1.60E-114	putative intracellular protease/amidase
26-20	-	-	-	gi152970293	99	7.20E-64	putative phosphotransferase system EIIC
44-48	-	-	-	gi152971363	97	2.70E-139	putative phosphatase/sulfatase
39-13	-	-	-	gi152971922	100	2.40E-71	putative fimbrial-like protein
11-34	-	-	-	gi152971945	59	7.50E-21	*metC* cystathionine beta-lyase
14-12	-	-	-	gi152970244	92	4.70E-34	major facilitator family transporter

*: WUBLASTN *e*-value <1×10^−5^.

The presence of previously described virulence factors in Kp342 encouraged virulence testing in an animal model. To evaluate the pathogenicity of Kp342, the ability of the strain to cause urinary tract and lung infection was investigated by use of mouse models. For comparison, the well-characterized clinical isolate C3091 was included in the study. Kp342 was able to cause urinary tract infections (UTI). Five out of six mice inoculated with strain Kp342 had infected bladders 3 days after inoculation, and the number of bacteria in infected bladders was similar to bladders of mice inoculated with the clinical strain C3091 (Table 3). Kp342 was also able to ascend to the kidneys, but at a level 28 times lower than the clinical strain, C3091 (*P* = 0.009).

**Table 3:** Infection of Kp342 and Clinical Strain *K. pneumoniae* C3091 in Mouse Urinary Tract Infection and Lung Infection Models.

Model	Tissue	Log CFU†	
		Kp342	C3091
UTI	Bladder	3.40 ± 0.72	3.94 ± 0.40
	Kidney*	2.43 ± 0.40	3.87 ± 0.45
Lung Infection	Liver	0.44 ± 0.44	1.99 ± 1.02
	Lung*	4.63 ± 0.41	6.32 ± 0.38
	Spleen	0	0.54 ± 0.35

**†:** Mean Log colony forming units (CFUs) recovered from each organ with standard error.

*: Statistically significant difference between Kp342 and C3091 infection at the 5% level as determined by Fisher's Least Significant Difference Test.

All mouse lungs were also infected with Kp342 two days after inhalation, but at a level 49 times less than C3091 (*P* = 0.015, Table 3) thus, it can be concluded that Kp342 causes lung infection, but at a significantly lower level than the infection level caused by C3901. Liver infection was detected in only one of the five mice following Kp342 inoculation compared with three of five mice infected with C3091. The spleen was infected in two of the five mice challenged with C3091 while none of the mice challenged with Kp342 were infected.

### Antibiotic Resistance

Kp342 has adapted or acquired many mechanisms of antibiotic resistance (Table 4). Considering this is a plant isolate with no contact with synthetic or man-made antibiotics, it is surprisingly multidrug resistant to all major drug families tested (Table 4). In contrast to many of the clinical multidrug-resistant isolates studied previously [71], which use a combination of point mutations and efflux mechanisms, Kp342 uses primarily efflux pumps and beta-lactamase genes to establish resistance to a variety of drugs. None of the classic antibiotic-resistance point mutations could be identified in *gyrA*, *gyrB*, *parC*, *parE*, *folP*, *rpoB* or 23S rRNA genes to account for quinolone, sulfonamide, rifampin and macrolide antibiotics. The genome encodes 4 bona fide beta-lactamase genes (KPK_1541, KPK_2697, KPK_2780 and KPK_2800), 7 genes in the metallo-beta-lactamase family and one beta-lactam resistance protein (*blr*, KPK_2388). Of these, KPK_2780 and KPK_2800 are identical and are part of a tandem duplication event, encompassing nucleotides 2834061-2850989 and 2850989-2867917. These two genes are nearly identical (98.6% identity) to the previously described chromosomally encoded class A beta-lactamase, SHV-1 [72]. Two additional CDSs, KPK_1541 and KPK_2697, are both predicted to encode class C beta-lactamases (matching COG1680). Kp342 encodes *ramA* (KPK_4028), a gene previously identified in *K. pneumoniae* that confers resistance to chloramphenicol, tetracycline, nalidixic acid, ampicillin, norfloxacin, trimethoprim and puromycin A when expressed in *E. coli* K12 [73]. Immediately upstream of this gene is *romA* (KPK_4029), which was originally isolated from *Enterobacter cloacae* as a gene that when expressed in *E. coli*, caused reduced expression of outer membrane proteins, resulting in a multiple drug resistance phenotype (quinolones, beta-lactams, chloramphenicol, and tetracycline) [74] that is independent of OmpF [75]. This gene has recently been shown to be adjacent to *ramA* in *K. pneumoniae* G340 during the sequencing of a tigecycline susceptible transposon mutant clone in *ramA* [76]. RamA has been shown to be a transcriptional activator similar to MarA (KPK_2759) [73] that increases expression of the RND-family multidrug efflux pump, AcrAB, (KPK_4236/ KPK_4237) in *K. pneumoniae* strain G340 [76].

**Table 4:** Kp342 Antibiotic Resistance Profile.

Drug Family	Drug (μg)	Phenotype	Diameter Zone of Inhibition (mm)			
				Interpretive Standards		
			Observed	Resistance	Intermediate	Sensitive
Aminocoumarin	Novobiocin (30)	Resistant	0	≤17		
Aminoglycoside	Gentamicin (10)	Intermediate	14		13–14	
	Kanamycin (30)	Sensitive	30			≥18
	Neomycin (30)	Resistant	9	≤12		
B-Lactam						
Cephalosporin	Cefotaxime (30)	Sensitive	25			≥23
	Cefoperazone (75)	Sensitive	24			≥21
	Cefazolin (30)	Sensitive	20			≥18
	Ceftriaxone (30)	Intermediate	15		14–20	
	Cefuroxime (30)	Intermediate	15		15–17	
	Cephalothin (30)	Resistant	14	≤14		
	Moxalactam (30)	Intermediate	22		15–22	
B-Lactam						
Penicillin	Ampicillin (10)	Resistant	0	≤15		
	Mezlocillin (75)	Intermediate	18		18–20	
	Penicillin (10)	Resistant	0			
	Piperacillin (100)	Intermediate	19		18–20	
	Ticarcillin (75)	Resistant	7	≤14		
Macrolide	Azithromycin (15)		10	*		
	Erythromycin (15)	Resistant	0	*		
Quinolone	Ciproflaxacin (5)	Intermediate	18		16–20	
	Nalidixic acid (30)	Resistant	8	≤13		
	Norfloxacin (10)	Resistant	0	≤12		
	Oxolinic acid (2)	Resistant	7	≤10		
Sulfonamide	Sulfisoxazole (0.25)	Resistant	8	≤12		
	Trimethoprim (5)	Resistant	0	≤10		
Tetracycline	Minocycline (30)	Resistant	8	≤14		
	Oxytetracycline (30)	Resistant	0	*		
	Tetracycline (30)	Resistant	10	≤14		
Other	Rifampin (5)	Resistant	0	*		

*: No interpretive standards given for the *Enterobacteriacaeae*.

In addition to the AcrAB-TolC multidrug efflux pump, Kp342 encodes several multidrug efflux pumps with top matches to well characterized loci, including EefABC (KPK_0055- KPK_0053) [77], OqxAB (KPK_1163/ KPK_1162) [78], MdtABCD (KPK_1639- KPK_1636) [79], and MacAB (KPK_3651/ KPK_3650) [80]. EefABC, from *Enterobacter aerogenes* (also a nosocomial pathogen), confers resistance to beta-lactams, quiolones, chloramphenicol and tetracyclines [77], while OqxAB from *E. coli* plasmid pOLA52, confers olaquindox and chloramphenicol [78]. The MdtABCD efflux pump from *E. coli* K12 provides resistance to novobiocon and deoxycholate [79], while the MacAB transport system, also from *E. coli* K12, is specific to macrolide antibiotics [80].

## Discussion

### Kp342 and MGH78578

Comparative genomic analyses between Kp342 and MGH78578 reveal an overall high degree of similarity between the genomes of the two strains; however, key differences in genetic content have been identified that are likely to be critical influences on their preferred host ranges and lifestyles (endophytic plant associations for Kp342 and presumably human pathogen for MGH78578). One major difference in metabolism is the ability of Kp342 to fix nitrogen which gives this organism an advantage for survival in nitrogen poor environments and favors plant associations [1].

Comparative analyses reveal differences in the distribution of fimbrial proteins important to surface attachment and effectors of signaling proteins such as the secondary messanger protein, c-di-GMP, which has been implicated in the regulation of a wide variety of bacterial traits and responses to environmental stimuli affecting biosynthesis of exopolysaccharides, formation of biofilms, and regulation of virulence genes [81]. Interactions between bacterial surface-associated structures such as polysaccharides and fimbriae are central to the types of bacterial adhesions and range of host cells to which attachment can be accommodated as well as to biofilm formation. Furthermore, the Kp342 HecA-like filamentous haemagglutinin (KPK_4110) protein was found to be unique to Kp342 in the 3-way comparison, with no orthologs in MGH78578. These results coupled with additional dissimilarities between Kp342 and MGH78578 in the distribution of regulatory content such as transcription and sigma factor regulators further suggest that there are important differences in the regulatory networks formed in Kp342 and MGH78578.

Variations in the distribution of genes related to Type IV and TypeVI secretory function may impact secretion of virulence factors or substances that promote interactions with plants. Finally, dissimilarities in transporter content were noted especially a greater expansion in ABC and MFS transporter families in Kp342 versus MGH78578 which may further effect the nature of compounds including those derived from plants that can be taken up or excreted by Kp342. Collectively, these divergences in nitrogen fixation, surface attachment, regulation and signaling, secretion and transport are likely to assert critical influences on the lifestyles of these two organisms despite generally similar gene content.

### Plant-Induced and Phytobacterial Only Genes

Comparative genome analyses have elucidated a set of genes in the Kp342 genome that share homology with known plant-induced genes (75) and a set of phytobacterial only genes (23 and 45) with inclusion or exclusion of MGH78578 as a non-phytobacterium, respectively. These gene sets provide important targets for future study to confirm their role in endophytic colonization by Kp342. Many of these plant-induced genes appear to be involved in the adaptation of bacteria to conditions within plant tissue, such as the limitation of amino acid and carbon source concentrations. The importance of amino acid biosynthesis in plant-microbe interactions is supported by the observation that *P. syringae* mutants impaired in the biosynthesis of some amino acids are unable to cause disease symptoms in tomato [82]. A TPS (KPK_A0226) with similarity to *hecA/B* of *Erwinia chrysanthemi* was identified in the phytobacteria only gene set, which may be involved in attachment to root surfaces. In *Pseudomonas putida* KT2440, a non-pathogenic, plant colonizing bacterium, a second TPS (*hlpAB*) was determined to be necessary for competitive root colonization [83]. The presence of this additional TPS operon important to colonization by a non-pathogenic plant associated bacteria gives support to the likelihood that the HecA/B homolog in Kp342 plays a prominent role in colonization and is a promising candidate for future study.

A suite of plant-induced genes have been implicated in bacterial response to oxidative stress and DNA damage due to plant defense responses, several of which are involved in DNA repair and have homologs in the Kp342 genome. For example, the Ada protein is required to activate the transcription of genes involved in adaptive response to DNA methylation damage caused by alkylating agents, and has also been shown to be activated by nitric oxide [84]–[86]. In addition, exonuclease (*uvrA*) functions in UV induced DNA repair, but has also been shown to participate in hydrogen peroxide and toxic chemical induced DNA damage repair, indicating that this gene may act to protect the bacteria against DNA-damaging compounds produced by plants [87]–[89].

These oxidative response genes are not limited to DNA repair pathways. In *E. coli*, fumarate hydratase as encoded by *fumC*, and which is part of the TCA cycle, is more highly expressed under conditions when superoxide radicals accumulate [90]. An alternative form of fumarate hydratase, encoded by *fum*A, is inactivated under oxidative conditions [90],[91]. Since an early plant defense response involves the increase of ROS, induction of oxidative stress related genes indicate the bacteria are actively evading this defense mechanism while colonizing plants. Acriflavine resistance protein A (*acrA*) is another stress response gene induced upon plant colonization, but does not appear to be triggered by oxidative stress. The product of this gene encodes a component of the AcrAB-TolC efflux pump that is important in toxic waste removal in bacteria and shows increased expression under stress conditions [92],[93].

The roles of the plant-induced gene set described here have been best characterized in plant pathogens. In contrast, the breadth and complexity of plant-bacterial associations beyond that of pathogens is reflected in the small number of phytobacteria-only genes suggesting that no one set of genes can collectively define each of these additional plant associated lifestyles. The role category distribution of the phytobacteria only gene sets determined in this analysis are dominated by hypothetical proteins or proteins of unknown function and genes related to nitrogen fixation. Completion of additional endophytic genomes will be necessary to determine if a core set of genes exclusive to or that defines an endophyte can be established. Further investigations including gene deletion studies in Kp342 will also be necessary to confirm if genes from either the plant-induced or phytobacteria-only gene sets also play a role in endophytic adaptation to plant tissue. Specifically, their actions in colonization and plant defense evasion need to be elucidated.

### Antibiotic Resistance

Considering Kp342 is not a clinical isolate, the intrinsic antibiotic resistance mechanisms must have been maintained for reasons in addition to antibiotic resistance, such as the removal of toxic plant metabolites, many of which have cyclic ring structures similar to antibiotics. For example, it has been noted previously in *E. coli* that there is a high association of organic solvent (cyclohexane) tolerance with fluoroquinolone resistance mutants, suggesting that bacteria may undergo adaptive responses to organic substances other than quinolones [94]. More recently, five of ten organic solvent-tolerant *K. pneumoniae* clinical isolates overexpressed AcrA and had deletions in the repressor *acrR* [71]. Resistance to commonly prescribed quinolones, such as ciprofloxacin, is enhanced when co-administered with salicylate [95],[96]. This phenomenon has been noted previously only in the context of co-treatments within a clinical setting and not in the natural environment. It seems reasonable to believe that the observed induction of antibiotic resistance by salicylate in *K. pneumoniae* [97],[98] is an unintended consequence of a natural response to the major plant signaling molecule salicylate, which is induced during bacterial pathogenesis and flower development [99].

### Pathogenicity

In the present study, the pathogenic potential of Kp342 was evaluated in mouse models of urinary tract and lung infection and compared to the clinical strain C3091. Kp342 was found to be as virulent as C3091 regarding the ability to infect the bladder, however although Kp342 was able to ascend to the kidneys, the number of bacteria in infected kidneys were significantly lower compared to C3091. In the lung infection model, all mice inoculated with Kp342 developed lung infections, although the number of bacteria in infected lungs was 49-fold lower compared to C3091. Dissemination of the infection to the liver was seen only in one of the five mice inoculated with Kp342, whereas in the group inoculated with C3091, infection of the liver or spleen was seen in three of the five mice. Compared to the clinical isolate C3091, the lower number of bacteria in infected kidneys and lungs and minor spreading of the infection to other organs indicates that Kp342 is potentially pathogenic, but is less virulent than typical clinical *K. pneumoniae* isolates.

### Conclusion

The core theme which defines an endophyte is an ability to live cooperatively within the interior of plant tissues without inducing, or effectively evading plant host defense systems. Comparative genomic analyses in combination with virulence studies in mice have revealed that Kp342 appears to achieve this balance in several ways. For instance, although multiple antibiotic resistance genes and virulence in animals were determined, in general, pathogenicity appears to be attenuated in this strain. Instead genome analyses revealed mechanisms favoring an association with plants. These include not only the capacity to fix nitrogen, but also the presence of metabolic pathways and transport systems well-suited to the recognition and catabolism of plant compounds such as the uptake and degradation of plant derived polysaccharides encompassing cellulosic and aromatic compounds, and survival against ROS and nitric oxide. Further, the distribution of genes essential to surface attachment, secretion, transport, and regulation and environmental signaling, varied between the Kp342 and MGH78578 genomes which may reveal critical divergences between the two strains influencing their preferred host ranges and lifestyles (endophytic plant associations for Kp342 and presumably human pathogen for MGH78578). The analysis reported here and completion of the entire Kp342 genome sequence should serve to catalyze future studies of this organism and provide a new lens through which to view and study the endophytic lifestyle which represents an important but less well-studied form of bacterial-host relationships and one that can potentially be utilized to enhance the growth and nutrition of important agricultural crops. In addition, these results will inform research on *Klebsiella* pathogenesis and development of plant-derived products and biofuels.

## Materials and Methods

### Strain Isolation and Verification

Kp342 was originally isolated as a nitrogen-fixing diazotroph from the interior stems of a greenhouse-grown, nitrogen-efficient *Zea mays* L. cv. CIMMYT 342 [9]. Strain 342 was verified as *K. pneumoniae* using 16S rRNA primers 27f and 1492r and biochemical tests on an API 20E system (Hazelwood, MO, USA) as described previously [9],[100]. *Klebsiella pneumonia* C3091 is a human clinical strain previously described [101],[102].

### Isolation and Purification of DNA for Library Production

Bacterial cultures were grown on LB medium followed by the isolation of genomic DNA using the FastDNA Kit from Q-BIOgene (Irvine, CA).

### Genome Sequencing

The genome of strain *K. pneumoniae* 342 was sequenced to closure by the whole random shotgun method [103]. Briefly, one small insert plasmid library (2–3 kb) and one medium insert plasmid library (10–15 kb) was constructed by random nebulization and cloning of genomic DNA. In the initial random sequencing phase, 8-fold sequence coverage was achieved from the two libraries (sequenced to 5-fold and 3-fold coverage, respectively). The sequences were assembled using the Celera Assembler [104]. Ordered scaffolds were generated by first aligning Kp342 contigs to the genome of *Escherichia coli* K12 using NUCMER [24], followed by BAMBUS [105]. All sequence and physical gaps were closed by editing the ends of sequence traces, primer walking on plasmid clones, and combinatorial PCR followed by sequencing of the PCR product.

An initial set of open reading frames (ORFs) that likely encode proteins was identified using GLIMMER [106], and those shorter than 90 base pairs (bp) as well as some of those with overlaps eliminated. A region containing the likely origin of replication was identified, and base pair 1 was designated adjacent to the *dnaA* gene located in this region [107]. ORFs were searched against a non-redundant protein database as previously described [108]. Frameshifts and point mutations were detected and corrected where appropriate. Remaining frameshifts and point mutations are considered authentic and corresponding regions were annotated as ‘authentic frameshift’ or ‘authentic point mutation’, respectively. The ORF prediction and gene family identifications were completed using the methodology described previously [108]. Two sets of hidden Markov models (HMMs) were used to determine ORF membership in families and superfamilies. These included 721 HMMs from Pfam v22.0 and 631 HMMs from the TIGR ortholog resource. TMHMM [109] was used to identify membrane-spanning domains (MSD) in proteins. Putative functional role categories were assigned internally as previously described [110].

The nucleotide sequence as well as the corresponding complete manually curated annotations for the closed genome of *K. pneumoniae* Kp342 were submitted to GenBank under GenomeProject ID #28471.

### Comparative Genomics

All predicted proteins from *K. pneumoniae* Kp342 were compared with data from other published microbial genomes using WUBLASTP (http://blast.wustl.edu)[111], against a database of 1,720,276 protein sequences composed of 473 finished bacterial, 163 eukaryotic, 29 archaeal, 26 mitochondrial, 3 nucleomorph, 18 plastid, and 35 viral chromosomal, as well as 303 plasmid accessions, encompassing 569 unique taxa. For binning of phytobacteria-specific protein sequences, unidirectional matches were scored that met the following prerequisites: an E-value of < = 1×10^−5^, > = 35% identity, and match lengths of at least 70% of the length of both query and subject. The complete genome of the clinical strain of *K. pneumoniae* MGH78578 was sequenced by the Genome Sequencing Center at Washington University School of Medicine and obtained from NCBI as RefSeq accession NC_009648. The average protein percent identity of Kp342 proteins compared to MGH78578 and *E. coli* K12 was calculated as previously described [103]. Transporter profiles were generated and compared using the TransportDB [112] as previously described [38],[39]. The generation of an ortholog matchtable, construction of the Venn diagram, and binning of relationships within the Venn diagram were completed as previously described [103] using the above mentioned database and cutoffs.

### Phytobacterial Analysis

An in-house PERL script was used to parse data from Kp342 CDSs searched against an in-house database of 1,720,276 protein sequences from 1050 accessions using WUBLASTP. In order to determine those CDSs found only in only phytobacteria, Kp342 proteins having a significant match to at least one phytobacterial protein but not to any other protein from any other organism in the database were obtained. This analysis was also repeated including MGH78578 in the non-phytobacterial group of genomes.

### Phylogenetic Analysis

The phylogenetic analyses were conducted using a system created to automatically generate and summarize phylogenetic trees for each protein for which phylogenetic analysis can be conducted in a genome. The APIS system was used to analyze the Kp342 genome as previously described [113]. Each phylogenetic tree is obtained by comparison of a query protein against a curated database of proteins from complete genomes using WUBLAST [114]. The full-length sequences of these homologs are then retrieved from the database and aligned using MUSCLE [115], and bootstrapped neighbor-joining trees are produced using QuickTree [116]. An advantage of QuickTree over other phylogenetic tree building programs is that it produces bootstrapped trees with meaningful branch lengths. Next, the inferred tree is midpoint rooted prior to automatic determination of the taxonomic classification of the organisms with proteins in the same clade as the query protein.

### Pathogenicity Testing

All animal experiments were conducted under the auspices of the Animal Experiments Inspectorate, the Danish Ministry of Justice.

### Mouse Model of Ascending Urinary Tract Infection (UTI)

Six- to eight-week-old female C3H inbred mice (Harlan Teklad, UK) were used. The UTI model has been previously described [117]. Briefly, anaesthetized mice were inoculated transurethrally with 50 µl bacterial suspension containing approximately 5×10^8^ CFU by use of plastic catheters. The catheter was carefully pushed horizontally through the urethral orifice until it reached the top of the bladder, and the bacterial suspension slowly injected into the bladder. The catheter was immediately removed and the mice subjected to no further manipulations until sacrifice. The mice were sacrificed 3 days after inoculation. Bacteria were recovered from the bladder and kidneys by homogenization in 1 ml 0.9% NaCl, serially diluted, and plated on McConkey agar (Oxoid).

### Mouse Lung Iinfection Model

An intranasal infection model was used as described [118],[119]. Six- to eight-week-old female NMRI outbred mice (Harlan Teklad, UK) were anaesthetized. The mice were hooked on a string by the front teeth and 50 µl bacterial suspension containing approximately 5×10^7^ CFU dripped onto the nares. The mice readily aspirated the solution and were left hooked on the string for 10 min before being returned to their cages. The mice were sacrificed 2 days after inoculation. Bacteria were recovered from the lungs, spleen and liver as described above in the UTI model.

### Statistical Analysis

Fisher's Least Significant Difference (LSD) test and the Mann-Whitney *U* test were used for statistical analysis of data from virulence studies. *P* values less than 0.05 were considered statistically significant.

### Antibiotic Susceptibility Testing

Antimicrobial Susceptibility Discs were obtained from Becton-Dickson BBL, with the exception of azithromycin and norfloxacin, which were obtained from Remel. Bacterial culture (5 ml) was grown for 4 hours at 37°C, adjusted to an OD_620_∼0.1, and swabbed onto Mueller-Hinton agar plates. Discs were dispensed four per plate and plates were incubated as directed by the manufacturer. Antibiotic sensitivity was determined by comparing zones of inhibition to interpretative standards as directed by the manufacturer.

## Supporting Information

Figure S1 Regional Display of the Nitrogen Fixation Genes in Kp342. The *nif* genes of Kp342 (C) was compared with the *nif* operon of *K. pneumoniae* from GenBank accession X13303 [21] (B) and the missing region in MGH78578 (A). The colors of the CDSs of Kp342 are by functional role category: protein synthesis; pink, regulatory functions; olive, energy metabolism; light gray, central intermediary metabolism; brown, biosynthesis of cofactors, prosthetic groups, and carriers; light blue, hypothetical proteins; crosshatch, transport and binding proteins; blue-green. The CDSs in A and B are not colored by role category. The shaded regions depict nucleotide percent identity using NUCMER (see key).
(0.28 MB EPS)

Figure S2 Phylogenetic Analysis of *celD* and *celK* of Kp342. Consensus Neighbor-joining trees are depicted using automated multiple alignments of *celD* (A) and *celK* (B) to homologs in other organisms. The thickness of the branches denotes percent occurrence of nodes among 100 bootstrap replicates.
(0.40 MB EPS)

Figure S3 Average Number of Usher Protein HMM Matches. A database of complete genomes was searched against PF00577, Fimbrial Usher protein. The x-axis displays the genus, while the y-axis denotes the average number of matches to PF00577 above the trusted cut off. The error bars show the standard deviation generated from multiple strains.
(0.13 MB PDF)

Table S1 Small Molecule Transporter Family Analysis of Kp342 Compared to *K. pneumoniae* MGH78578, *E. coli*, and Representative Soil and Plant-associated Bacteria.
(0.22 MB XLS)

Table S2 Site-Specific Integrated Elements Found in the Genome of Kp342.
(0.04 MB XLS)

Table S3 Orthologous Protein Matches to Kp342.
(1.20 MB XLS)

Table S4 Proteins Shared Only between the *Klebsiella* Strains 342 and MGH78578 from the Comparison of the *K. pneumoniae* 342, *K. pneumoniae* MGH78578, and *E. coli* K12 Genomes.
(0.46 MB XLS)

Table S5 *K. pneumoniae* 342-Specific Proteins from the Comparison of the *K. pneumoniae* 342, *K. pneumoniae* MGH78578, and *E. coli* K12 Genomes.
(0.21 MB XLS)

Table S6 *K. pneumoniae* MGH78578-Specific Proteins from the Comparison of the *K. pneumoniae* 342, *K. pneumoniae* MGH78578, and *E. coli* K12 Genomes.
(0.12 MB XLS)

Table S7 Proteins Shared Only between *K. pneumoniae* 342 and *E. coli* K12 from the Comparison of the *K. pneumoniae* 342, *K. pneumoniae* MGH78578, and *E. coli* K12 Genomes.
(0.07 MB XLS)

Table S8 Proteins Shared Only between *K. pneumoniae* MGH78578 and *E. coli* K12 from the Comparison of the *K. pneumoniae* 342, *K. pneumoniae* MGH78578, and *E. coli* K12 Genomes.
(0.05 MB XLS)

Table S9 Kp342 Proteins Shared only with Phytobacteria.
(0.04 MB XLS)

Table S10 Kp342 BLASTP Matches to Known Plant-Induced Proteins.
(0.07 MB XLS)

Table S11 Identification of Signature-tagged *K. pneumoniae* KPPR1 Mutants Failing Recovery from Lungs or Spleens of Infected Mice [69] in Kp342 and MGH78578.
(0.06 MB XLS)

Table S12 Identification of Signature-tagged *K. pneumoniae* C3091 Mutants Failing Recovery from Gastrointestinal and Urinary Tract Infection Mouse Models [68] in Kp342 and MGH78578.
(0.04 MB XLS)

Table S13 Identification of *K. pneumoniae* CG43 Genes from IVET [70] in Kp342 and MGH78578.
(0.04 MB XLS)

Text S1 A General Synopsis of Central Intermediary and Energy Metabolism, Including Sulfur and Phosphorous Metabolism and Electron Transport.
(0.05 MB DOC)
