# Supplementary figures and images for: Complete Genome Sequence of the N2-Fixing Broad Host Range Endophyte Klebsiella pneumoniae 342 and Virulence Predictions Verified in Mice
Source: PLoS Genet. 2008 Jul 25;4(7):e1000141. doi: 10.1371/journal.pgen.1000141 (PMC2453333; doi:10.1371/journal.pgen.1000141)

Average Number of Usher Protein HMM Matches

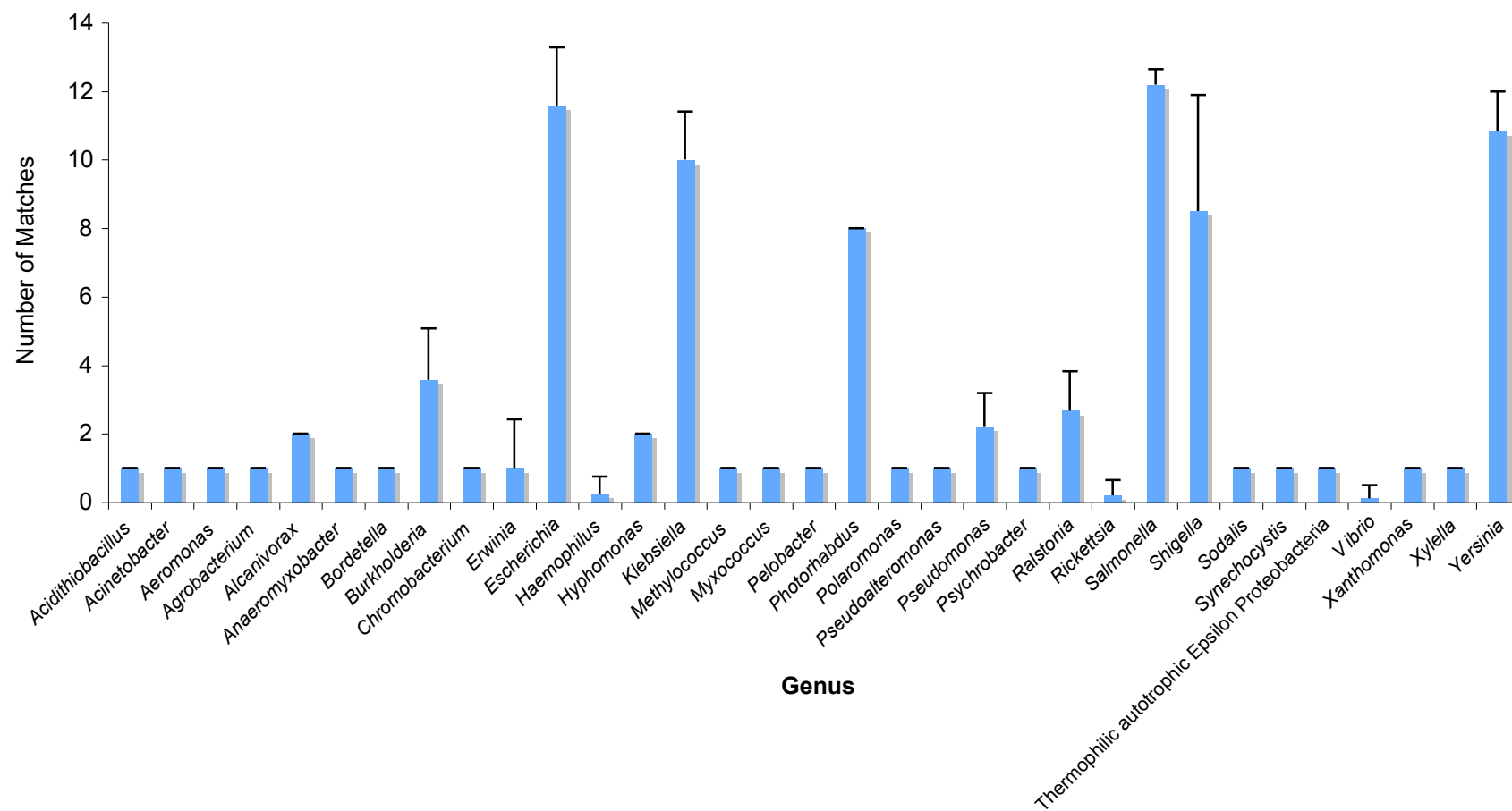

Supplement: Figure S3 — Average Number of Usher Protein HMM Matches. A database of complete genomes was searched against PF00577, Fimbrial Usher protein. The x-axis displays the genus, while the y-axis denotes the average number of matches to PF00577 above the trusted cut off. The error bars show the standard deviation generated from multiple strains. (0.13 MB PDF) [file pgen.1000141.s003.pdf]
