# Supplementary material for: Complete Genome Sequence of the N2-Fixing Broad Host Range Endophyte Klebsiella pneumoniae 342 and Virulence Predictions Verified in Mice
Source: PLoS Genet. 2008 Jul 25;4(7):e1000141. doi: 10.1371/journal.pgen.1000141 (PMC2453333; doi:10.1371/journal.pgen.1000141)
Supplement: Text S1 — A General Synopsis of Central Intermediary and Energy Metabolism, Including Sulfur and Phosphorous Metabolism and Electron Transport. (0.05 MB DOC) [file pgen.1000141.s017.doc]

**Text S1. A General Synopsis of Central Intermediary and Energy Metabolism, Including Sulfur and Phosphorous Metabolism, Fermentation, Additional Aromatic Compound Degradation and Electron Transport**

**Central Intermediary Metabolism**

Genome analyses of Kp342 revealed a robust central intermediary and energy metabolism typical of most enteric bacteria (and free-living heterotrophs). Of importance to central intermediary metabolism was the determination in the Kp342 genome of the presence of an F1/FO ATPase complex, glycolytic pathway, complete TCA cycle, glyoxylate shunt, and Entner-Doudoroff and pentose phosphate pathways. An ability to form the critical central intermediary metabolite, acetyl-CoA from pyruvate could occur oxidatively via pyruvate dehydrogenase and anaerobically using pyruvate formate lyase. A third possibility was also revealed through the presence of a putative pyruvate:flavodoxin oxidoreductase (KPK_3026) capable of electron transfers from pyruvate to flavodoxin during fermentation reactions.

Sulfur and Phosphorous

Kp342 possesses standard metabolic processes for the assimilation and internal cycling of sulfur and phosphorous. The presence of an assimilatory sulfate reduction pathway including the presence of ATP sulfurylase, (KPK_1009- KPK_1011), phosphoadenosine phosphosulfate reductase (PAPs reductase KPK_1006) and sulfite reductase (KPK_1005- KPK_1004) was determined. The Kp342 genome also contains at least five members of a paralogous family of sulfatases responsible for catalyzing the release of sulfur from organic compounds generally through the hydrolysis of a sulfuric ester bonds (KPK_1536, KPK_0202, KPK_5351, KPK_3764, KPK_3725). Genome analyses revealed the presence of the Pho transport system for phosphorous uptake and the phosphonate C-P lyase system responsible for the uptake of alkylphosphonates (KPK_5173- KPK_5168). Further, the Kp342 genome maintains CDSs necessary for polyphosphate metabolism (exopolyphosphatase, KPK_1303, polyphosphate kinase KPK_1304). Polyphosphates are linear polymers consisting of hundreds of orthophosphate residues linked by phosphoanhydride bonds that have a variety of physiological functions, including creation of a phosphate reservoir and more recently has been linked to a possible role in resistance to heavy metals (Remonsellez et al. 2006).

**Energy Metabolism**

Electron Transport and Electron Acceptors

Overall, Kp342 has a versatile and complex energy metabolism and is capable of oxygenic and anaerobic respiration as well as a variety of fermentative reactions. As part of an oxidative electron transport, Kp342 possesses the heme-copper containing bo-type cytochrome oxidase (KPK_4606- KPK_4294), which has a low oxygen affinity and is preferentially utilized under high oxygen conditions. In contrast, the high oxygen affinity heme protein cytochrome bd complex, for which the Kp342 genome contains three copies (CydA KPK_2422, KPK_3338, KPK_3834 and CydB KPK_3833, KPK_2421, KPK_3336), is synthesized during microaerophilic conditions. The Kp342 genome was determined to encode genes for all important classes of electron carriers, including both ubiquinone, used largely during aerobic respiration, and menaquinone used preferentially during anaerobic conditions. Furthermore, several flavoproteins and Fe-S proteins were identified, although only one c-type cytochrome (KPK_2251) was determined.

Under anaerobic conditions, Kp342 is capable of respiration using several alternative electron acceptors. The ability to use nitrate or dimethyl sulfoxide (DMSO) was confirmed by the presence of two copies of respiratory nitrate reductase (KPK_2476- KPK_2473, KPK_2098- KPK_2094) and one DMSO reductase (KPK_3634- KPK_3632), respectively. A second putative copy of DMSO reductase (KPK_2877- KPK_2875) was also identified by genomic analyses, suggesting that additional sulfoxide or N-oxide compounds may serve as alternate electron acceptors for Kp342. The genome also possesses a fumarate reductase (KPK_5118- KPK_5115) which may allow fumarate to be used not only in mixed acid fermentations but also as a terminal electron acceptor during growth under anaerobic conditions. In this reaction the electron donor is reduced by menaquinone while generating a proton gradient in a scalar mechanism by utilizing two protons from the cytoplasm (quinone loop).

Kp342 is also capable of producing both proton and sodium ion currents as part of its energy metabolism. This is reflected through the presence of the NADH dehydrogenase complex (KPK_1471-KPK_1483) responsible for conserving energy from redox reactions in a proton gradient (for every two electrons transferred, four hydrogen ions are translocated across the cytoplasmic membrane) and the Na^+^-translocating NADH quinone reductase complex. The latter complex is central to ability of Kp342 to couple the fermentation of carbon intermediates such as citrate to a Na^+^ ion current in conjunction with the Na^+^-dependent decarboxylation of oxaloacetate by oxaloacetate decarboxylase under anaerobic conditions. Two copies of the oxaloacetate decarboxylase complex were identified. The Kp342 genome also contains an Rnf-type electron transport complex (KPK_2384- KPK_2384) likely utilized as an NADH oxidoreductase responsible for shuttling electrons to nitrogenase during nitrogen fixation.

Fermentation

In addition to the aforementioned citrate fermentation, the Kp342 genome possesses a large complement of genes encoding enzymes capable of participating in a variety of mixed fermentative reactions. Mixed acid fermentations can occur through the catabolism of phosphoenolpyruvate which can either be shunted to oxaloacetate and ultimately succinate or to pyruvate with possible end-products of lactate, acetate and ethanol as well as catabolism to formate and ultimately carbon dioxide and hydrogen gas. Genome analyses also suggest the ability to catalyze the acetoin by 2,6-dichlorophenolindophenol-dependent cleavage (KPK_4460, KPK_4461, KPK_4462, KPK_4463) into acetate and acetaldehyde for which homologs in MGH78578 are not present. Kp42 can also ferment pyruvate to acetoin (KPK_2271-KPK_2270), although no butanediol dehyrogenase capable of metabolizing acetoin to an end-product of butanediol was found.

Formate dehydrogenase

The production of formate is a key carbon intermediary in Kp342. The ability to further metabolize this compound is noted in the Kp342 genome through the presence of the three major forms of formate dehydrogenase similar to those described in other enteric bacteria. The first form functions by allowing the use of formate as major electron donor during anaerobic respiration, when nitrate is used as electron acceptor (KPK_2491-KPK_2489). The second form (KPK_5492-KPK_5494) is critical to facilitating a rapid transition from aerobic to an anaerobic environment. Subunits of a third form are also present based on genome analyses and appear to encode a formate hydrogenlyase, which consists of the action of two enzyme complexes, formate dehydrogenase of which the Kp342 genome possesses two copies, (KPK_2240, KPK_5187) and a hydrogenase complex (KPK_1066-KPK_1061) that together are responsible for catalyzing a non-energy conserving reaction in which carbon dioxide and hydrogen gas is released from formate.

Alternative Dioxygenases for Aromatic Ring Cleavage

Additional ring hydroxylating dioxygenases were identified in the Kp342 genome although their substrate specificities or the pathways in which they participate are less well known. For example, an alternative dioxygenase, the 4,5-protocatechuate dioxygenase whose catabolic pathway is less understood but is thought to produce pyruvate and oxaloacetate as end products (Hara et al. 2003) may be present in Kp342. The alpha subunit of the 4,5-protocatechuate dioxygenase was identified (KPK_0043). Other lignin degrading enzymes that may function in a 4,5- protocatechuate dioxygenase pathway (Hara et al. 2003) include a two gene cluster co-localized on the main chromosome with the alpha subunit of the 4,5-protocatechuate dioxygenase (KPK_0043), consisting of a putative 4-carboxy-4-hydroxy-2-oxoadipate aldolase (KPK_0045) and an uncharacterized protein homologous to one involved in the degradation of polyaromatic hydrocarbon fluorene by *Sphingomonas* sp. (KPK_0046).

In addition, the Kp342 genome possesses a dioxygenase with a Lignin B catalytic domain, whose substrate specificity is unknown (KPK_0670). Further, a 2,3-dihydroxyphenylpropionate 1,2-dioxygenase (KPK_2202) was identified which in addition to acting on 2,3-dihydroxyphenylpropionate has also been shown in *E. coli* to catalyze meta-ring cleavage of 3-methylcatechol and catechol, although with reduced catalytic efficiency (Bugg 1993).

**References for Supplemental Text**

Remonsellez F, Orell A, Jerez CA (2006). Copper tolerance of the thermoacidophilic archaeon *Sulfolobus metallicus*: possible role of polyphosphate metabolism. Microbiology 152: 59-66.

Hara H, Masai E, Miyauchi K, Katayama Y, Fukuda M (2003) Characterization of the 4-carboxy-4-hydroxy-2-oxoadipate aldolase gene and operon structure of the protocatechuate 4,5-cleavage pathway genes in *Sphingomonas paucimobilis* SYK-6. Journal of bacteriology 185: 41-50.

Bugg TD (1993) Overproduction, purification and properties of 2,3-dihydroxyphenylpropionate 1,2-dioxygenase from Escherichia coli. Biochimica et biophysica acta 1202: 258-264.
